# Comparative analysis of fluoride-based and natural mouthwashes on NiTi orthodontic wire surface integrity

**DOI:** 10.2340/biid.v12.45036

**Published:** 2025-12-17

**Authors:** Gabriela Mishell Salinas Sánchez, Marjory Elizabeth Vaca Zapata, Mauricio Aguirre Balseca, Karina Maria Salvatore Freitas, Stalin Wladimir Tamami Tualombo

**Affiliations:** a Department of Health Sciences, University of the Hemispheres, Quito, Ecuador; b Department of Orthodontics, Ingá University Center Uningá, Maringá, Brazil

**Keywords:** Orthodontic wires, mouthwashes, surface roughness, nickel-titanium alloys, corrosion

## Abstract

**Objective:**

To evaluate in vitro the effect of two commercial mouthwashes, Encident Brackets^®^ (fluoride- and chlorhexidine-containing) and BambooSmile^®^ (natural formulation) on the surface roughness of nickel–titanium (NiTi) orthodontic archwires.

**Materials and methods:**

Thirty rectangular NiTi archwire segments (0.019 × 0.025”, Orthometric) were divided into three groups (*n* = 10 each): control, Encident Brackets^®^, and BambooSmile^®^. Samples were pre-immersed in artificial saliva for 24 h, then exposed for 1.5 h to the respective solutions, simulating 30 days of clinical use. Surface roughness (Rz) was measured before and after immersion using a Marsurf PS10 profilometer. Statistical analysis included Student’s *t*-test and ANOVA (*p* < 0.05).

**Results:**

Both mouthwashes significantly increased surface roughness compared to baseline (*p* < 0.05). Mean Rz values rose from 0.798 to 2.208 μm in the Encident Brackets^®^ group and from 0.782 to 2.085 μm in the BambooSmile^®^ group. However, no significant differences were observed between the two experimental groups after treatment (*p* > 0.05).

**Conclusions:**

Exposure to both conventional and natural mouthwashes resulted in significant surface alterations of NiTi archwires. Although Encident Brackets^®^ produced slightly higher roughness values, its effect was comparable to BambooSmile^®^. These findings highlight the importance of considering mouthwash composition during orthodontic treatment, as increased surface roughness may compromise sliding mechanics, favor bacterial adhesion, and affect periodontal health. Further in vivo studies are recommended to validate these results under clinical conditions.

## Introduction

Orthodontic treatment relies on the use of biocompatible materials and mechanical systems to correct malocclusions, optimize occlusal function, and improve esthetics [1]. Among these materials, nickel-titanium (NiTi) archwires play a central role due to their unique superelasticity and shape memory, allowing continuous light forces that improve treatment efficiency and patient comfort [2, 3]. However, the long-term clinical performance of NiTi wires depends on their ability to maintain structural and chemical integrity under complex oral conditions [4, 5].

The oral cavity represents a dynamic and challenging environment, characterized by fluctuations in pH, temperature, enzymatic activity, and microbial colonization, as well as continuous exposure to chemical agents such as fluorides and antiseptic compounds [4, 6]. These factors may compromise the protective titanium oxide (TiO₂) layer on NiTi surfaces, thereby increasing the risk of corrosion, ion release, and surface roughening [7, 8]. Clinically, surface deterioration reduces sliding mechanics by increasing friction at the bracket–wire interface, prolongs treatment time, facilitates bacterial adhesion, and may promote periodontal inflammation [9, 10].

Fluoride-containing mouthwashes, while effective in caries prevention, are known to accelerate corrosion of metallic orthodontic appliances, leading to greater ion release and rougher surfaces [11–13]. Similarly, chlorhexidine, widely used for its antimicrobial activity, may alter surface characteristics when used chronically [14]. In contrast, natural alternatives without fluoride or chlorhexidine, often formulated with plant-derived compounds and essential oils, have been proposed as less aggressive options, though their effects on NiTi alloys remain poorly understood [15–18].

Encident Brackets^®^ is a conventional orthodontic mouthwash containing sodium fluoride, chlorhexidine digluconate, xylitol, and chamomile extract. Its formulation targets caries and gingival inflammation but may enhance corrosion risk due to its fluoride and chlorhexidine content. Other commercial products and natural rinses containing xylitol and chamomile support that such ingredient combinations are plausible [19]. BambooSmile^®^, in contrast, is a fluoride- and chlorhexidine-free natural product, formulated with sodium bicarbonate, tea tree oil, rosemary, thyme, lemon, and plant extracts, whose antimicrobial and anti-inflammatory properties have been documented in the literature [15, 16, 20]. Comparative studies indicate that fluoridated or alcohol-based rinses produce significantly higher surface alterations in orthodontic alloys than natural formulations [13, 21].

Surface roughness is a critical parameter for assessing the corrosion resistance and clinical performance of NiTi wires. The average surface roughness (Ra) and related rugosimetric metrics provide reliable quantitative measures of surface degradation, which directly influence frictional mechanics, ion leaching, and bacterial adhesion in the oral environment [22–24].

Given the widespread use of mouthwashes in orthodontic patients and the lack of comparative data between conventional and natural formulations, it is essential to clarify their effects on the surface integrity of NiTi archwires. This way, the aim of this study was to evaluate in vitro the effect of two mouthwashes on the surface roughness of NiTi wires: Encident Brackets^®^ (containing fluoride and chlorhexidine) and BambooSmile^®^ (natural, without fluoride or chlorhexidine).

## Material and methods

This was an in vitro experimental comparative study designed to evaluate the effect of two commercial mouthwashes, Encident Brackets^®^ and BambooSmile^®^, on the surface roughness of orthodontic NiTi rectangular wires (0.019 × 0.025 inches, 2 cm in length, superelastic; Orthometric, Marília, Brazil).

The a priori sample size was calculated for the primary comparison of post-exposure surface roughness (Rz) between the two experimental groups using a two-tailed independent-samples *t* test (α = 0.05). Based on pilot data that showed a substantial increase in roughness after exposure, a large standardized effect size (Cohen’s d = 1.05) was assumed. Under these parameters, 10 specimens per group provide a power (1 − β) = 0.80 to detect statistically significant differences (G*Power 3.1.9.7; test family: *t* tests; statistical test: means – difference between two independent means).

For within-group pre- and post-exposure comparisons, the same sample size (*n* = 10) also yields > 80% power to detect a large paired effect (dz ≥ 1.0) at α = 0.05. Therefore, the enrolled *n* = 10 per group was considered adequate for both inter- and intra-group analyses.

The study was conducted in accordance with the criteria of American Dental Association (ADA) Specification No. 116: Oral Rinses (ISO 16408:2004, MOD) and ADA Standard No. 32: Orthodontic Wires (ISO 15841:2014, MOD). Prior to the main study, a pilot test with 10 wire segments was performed, showing increased surface roughness after exposure to the tested solutions, thereby justifying the experimental design.

A total of 30 new, factory-sealed NiTi wire segments were included. All wires were verified for dimensions and integrity prior to testing. Artificial saliva (Saliv^®^, Lamosan Laboratories) was used to simulate intraoral conditions, while Encident Brackets^®^ (fluoride- and chlorhexidine-containing) and BambooSmile^®^ (fluoride- and chlorhexidine-free, plant-based formulation) served as the experimental agents. Double-distilled water (Sanderson) was used for rinsing at the end of each immersion cycle.

**Figure 1:**
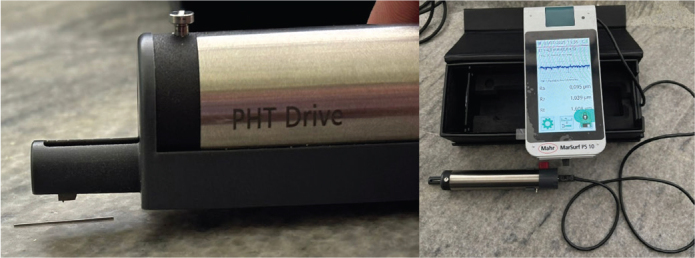
Surface roughness tester.

**Figure 2:**
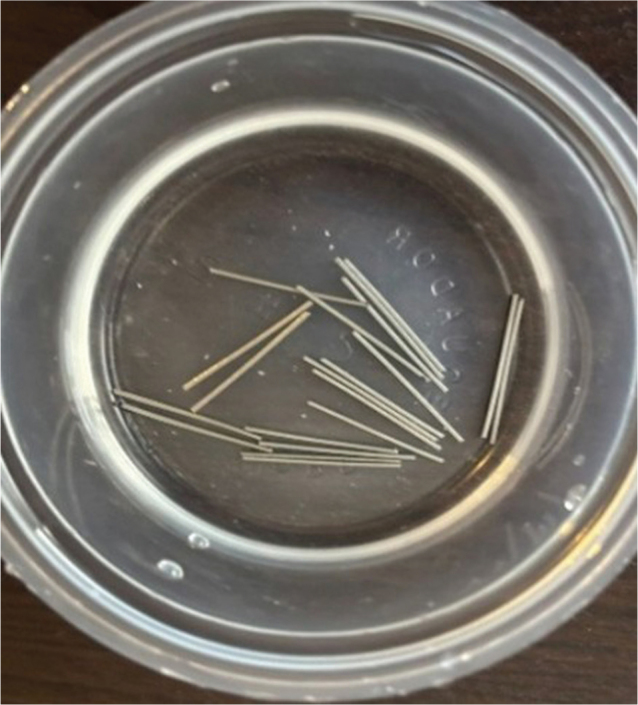
Immersion test in artificial saliva.

Baseline measurements of surface roughness were recorded for all 30 samples using a Marsurf PS10 profilometer with a diamond stylus, operating at 0.05 mm/s and recording up to 9,600 points to detect microscopic surface irregularities (Figure 1). After baseline evaluation, all samples were immersed in 5 mL of artificial saliva for 24 h at room temperature to simulate prior exposure to the oral environment (Figure 2).

**Figure 3:**
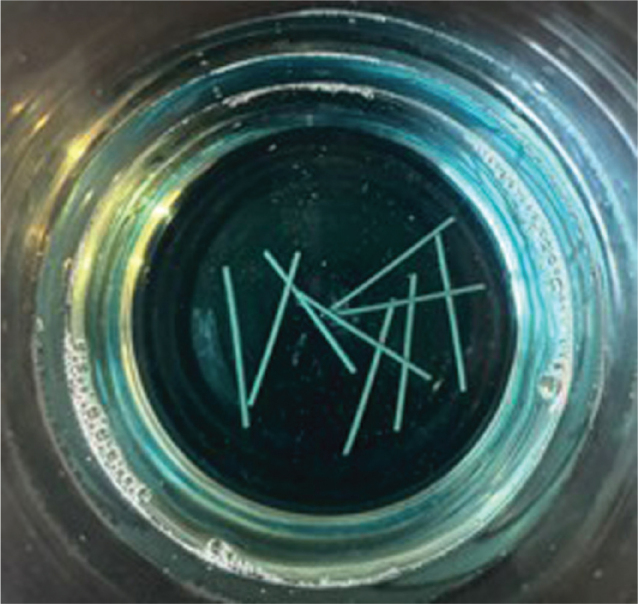
Immersion test in Encident Brackets mouthwash.

**Figure 4:**
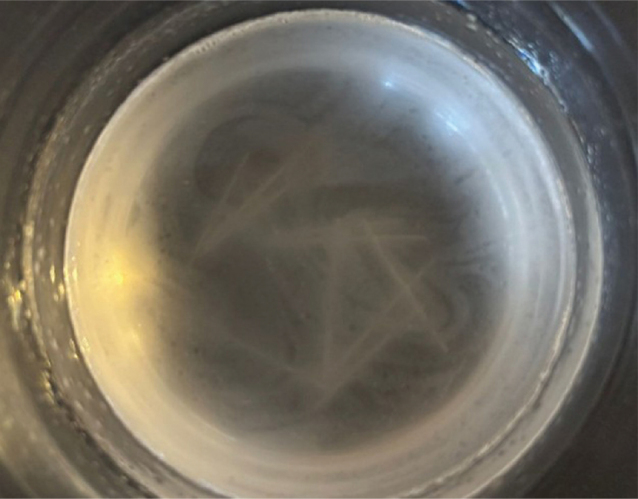
Immersion test in Bamboo Smile mouthwash.

Subsequently, the specimens were randomly divided into three groups:

- Control group (*n* = 10): no exposure to mouthwash.
- Encident Brackets^®^ group (*n* = 10): immersion in 10 mL of Encident Brackets^®^ mouthwash (Figure 3).
- BambooSmile^®^ group (*n* = 10): immersion in 10 mL of BambooSmile^®^ mouthwash (Figure 4).

To reproduce clinical use, equivalent to three daily rinses of 1 min over 30 days [25], an accumulated exposure time of 90 min per sample was established. For practical standardization, each sample underwent a single continuous immersion of 1.5 h, with the solution renewed every 10 min, resulting in nine solution changes throughout the protocol [26].

After treatment, the specimens were rinsed with double-distilled water to remove any residues and repositioned on a flat surface for post-exposure analysis. Final roughness measurements were performed using the same profilometer under identical conditions.

Surface roughness was quantified using the parameter Rz (average maximum peak-to-valley height) because it is more sensitive to localized irregularities than (average surface roughness), providing a more precise assessment of critical surface defects that may compromise the mechanical and biological performance of orthodontic wires. Lower values indicated smoother surfaces, while higher values reflected increased surface roughness and degradation.

### Statistical analysis

Normality tests of Shapiro-Wilk and Kolmorogov-Smirnov were applied, demonstrating normal distribution, allowing parametric tests. Dependent tests were used to compare the initial and final surface roughness in each group, and independent *t* tests were performed to compare the groups (Encident Brackets and BambooSmile) at initial and final evaluations. Results were considered significant for *p* < 0.05.

## Results

Before immersion, the baseline surface roughness (Rz) values did not differ significantly among groups, confirming sample homogeneity (Encident Brackets^®^ = 0.798 ± 0.171 μm; BambooSmile^®^ = 0.782 ± 0.124 μm; *p* = 0.771).

**Figure 5:**
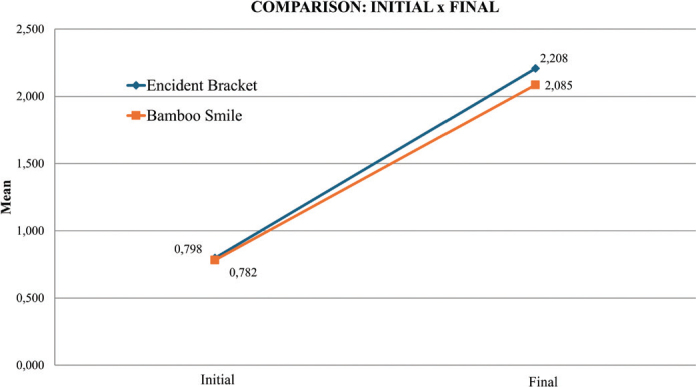
Intragroup comparison of initial x final stages for Encident Brackets and Bamboo Smile.

**Figure 6:**
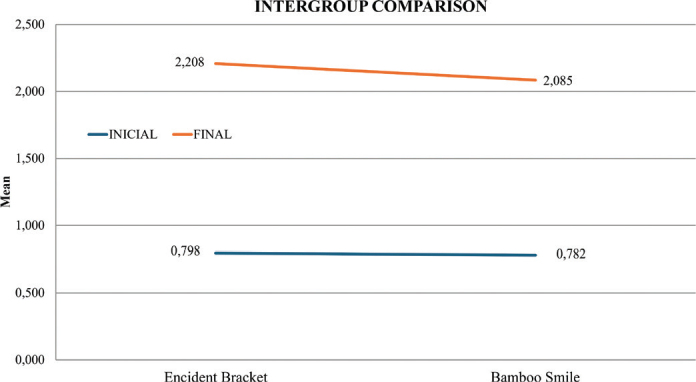
Comparison between Encident Brackets and Bamboo Smile.

For the Encident Brackets^®^ group, the mean initial surface roughness (Rz) was 0.798 ± 0.171 μm. After exposure, roughness increased significantly to 2.208 ± 0.193 μm (*p* < 0.001, paired *t* test) (Figure 5). Similarly, the BambooSmile^®^ group showed an initial mean roughness of 0.782 ± 0.124 μm, which rose to 2.085 ± 0.438 μm after treatment (*p* < 0.001) (Figure 6). Thus, both mouthwashes produced a statistically significant increase in surface roughness compared with baseline (Table 1).

**Table 1:** Comparison of initial and final measurements in each group (dependent *t* tests).

Groups	*N*	Mean (Rz)	SD	*p*
**Encident Brackets**				
Initial	15	0.798	0.171	0.000
Final	15	2.208	0.193	
Total	30	0.790	0.147	
**BambooSmile**				
Initial	15	0.782	0.124	0.000
Final	15	2.085	0.438	
Total	30	2.147	0.338	

When comparing the two groups, no statistically significant differences were detected either at baseline (*p* = 0.771) or after exposure (*p* = 0.329). This indicates that although both rinses increased roughness, their effects on NiTi surfaces were comparable (Table 2).

**Table 2:** Comparison between Encident Brackets and BambooSmile at initial and final evaluations (independent *t* tests).

Groups	*N*	Mean (Rz)	SD	*P*
**Initial**				
Encident Brackets	15	0.798	0.171	0.771
Bamboo Smile	15	0.782	0.124	
Total	30	0.790	0.147	
**Final**				
Encident Brackets	15	2.208	0.193	0.329
Bamboo Smile	15	2.085	0.438	
Total	30	2.147	0.338	

From a clinical perspective, this consistent increase in roughness suggests that both conventional (fluoride- and chlorhexidine-containing) and natural formulations may alter the surface integrity of NiTi wires, potentially influencing their biomechanical behavior.

**Figure 7:**
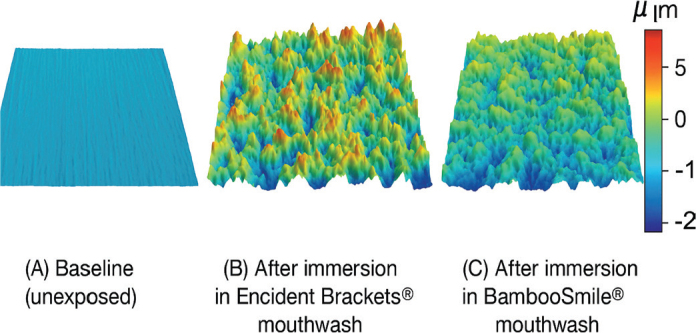
Representative 3D profilometric images of NiTi orthodontic wire surfaces: (A) baseline (unexposed), (B) after immersion in Encident Brackets^®^ mouthwash, and (C) after immersion in BambooSmile^®^ mouthwash. The images demonstrate the marked increase in surface irregularities after exposure, consistent with the quantitative roughness values reported.

Representative 3D profilometric surface maps of NiTi wire segments before and after immersion in each solution are shown in Figure 7, illustrating the increase in surface irregularities consistent with the roughness measurements obtained.

## Discussion

The corrosion resistance of nickel–titanium (NiTi) orthodontic wires is a critical determinant of their long-term mechanical performance and biocompatibility. In this study, both Encident Brackets^®^ (fluoride- and chlorhexidine-containing) and BambooSmile^®^ (natural, fluoride- and chlorhexidine-free) mouthwashes produced a statistically significant increase in surface roughness (*p* < 0.05). This finding underscores the vulnerability of NiTi alloys to chemical challenges in the oral environment, regardless of whether the formulation is conventional or natural.

Previous studies have consistently demonstrated that fluoride-containing agents can compromise the protective TiO₂ layer on NiTi surfaces, triggering localized corrosion, pitting, and fissures. Barrett et al. [11] and Eliades et al. [8] reported that fluoride exposure disrupts the passive oxide layer, leading to increased ion release and surface roughening. Similarly, Chantarawaratit and Yanisarapan [7] and Farrag et al. [21] confirmed that fluoride and chlorhexidine synergistically accelerate corrosion and alter surface morphology. These processes not only impair wire integrity but also promote bacterial adhesion and plaque accumulation, [6, 22] with potential consequences for periodontal health.

The present study aligns with these observations: surface roughness (Rz) increased from 0.798 to 2.208 μm after Encident Brackets^®^ exposure, and from 0.782 to 2.085 μm after BambooSmile^®^. While Encident Brackets^®^ induced slightly greater alterations, the difference between groups was not statistically significant (*p* > 0.05). This suggests that although fluoride and chlorhexidine are well-recognized corrosive agents, natural formulations are not free from risk. Components such as sodium bicarbonate and essential oils may also modify NiTi surfaces under prolonged exposure. Yıldırım et al. [13] similarly observed that even fluoride-free rinses can increase surface roughness, albeit to a lesser degree.

From a clinical perspective, the increase in surface irregularities is highly relevant. Roughened NiTi surfaces are associated with higher friction at the bracket-wire interface, [9, 10] reducing the efficiency of sliding mechanics and potentially prolonging treatment duration. Additionally, increased roughness creates niches for bacterial retention, enhancing the risk of gingival inflammation and periodontal compromise. [22] For patients with nickel hypersensitivity, surface degradation may further exacerbate metal ion release, posing additional biological risks [12, 27].

It is noteworthy that BambooSmile^®^, despite being marketed as a natural and eco-friendly alternative, produced surface alterations comparable to those of Encident Brackets^®^. Essential oils and plant-derived compounds can exhibit antimicrobial and anti-inflammatory effects [15–17], yet their long-term impact on orthodontic alloys remains underexplored. The current findings highlight that ‘natural’ does not necessarily equate to ‘neutral’ in terms of material compatibility.

The simulated exposure protocol, equivalent to 30 days of clinical use [25], provides a controlled model for evaluating surface changes. However, extrapolation to in vivo conditions must be cautiously, as oral environment includes variable salivary flow, pH fluctuations, thermal cycling, and masticatory forces [4]. These additional factors may exacerbate or mitigate corrosion dynamics, influencing clinical outcomes.

Taken together, the results confirm that NiTi wires are highly susceptible to surface degradation upon exposure to both conventional and natural mouthwashes. This reinforces the need for clinicians to carefully consider the type of oral hygiene adjuncts prescribed during orthodontic treatment. Future research should evaluate long-term in vivo effects, alternative alloy coatings, and the development of mouthwash formulations specifically designed to minimize adverse interactions with orthodontic materials.

From a clinical standpoint, the findings of this study underscore the importance of evaluating not only the antimicrobial efficacy but also the material compatibility of mouthwashes prescribed during orthodontic treatment. Even short-term exposure leads to significant surface alterations in NiTi wires, which can increase sliding resistance, prolong treatment time, and facilitate bacterial adhesion at the bracket–wire interface. Clinicians should therefore exercise caution when recommending fluoride- or chlorhexidine-based rinses for patients with fixed appliances and consider limiting their use to short periods or alternating with neutral or plant-based formulations.

Future research should aim to simulate more realistic intraoral conditions, including variable pH, temperature changes, and masticatory forces, to better approximate clinical performance. Long-term *in vivo* trials are needed to evaluate corrosion and ion release under continuous use, while advanced microscopy and nano-mechanical testing could further elucidate microstructural changes. Moreover, developing protective surface coatings or novel mouthwash formulations with reduced corrosive potential may represent promising strategies to preserve the integrity of orthodontic alloys.

## Conclusion

This study demonstrated that exposure to both Encident Brackets^®^ and BambooSmile^®^ mouthwashes significantly increased the surface roughness of NiTi orthodontic archwires. Although Encident Brackets^®^ produced slightly higher roughness values, the difference between groups was not statistically significant. These findings suggest that both conventional and natural formulations may compromise the surface integrity of NiTi wires, potentially increasing friction, bacterial adhesion, and periodontal risks. Therefore, clinicians should carefully consider the choice of mouthwash in orthodontic patients, and future in vivo studies are needed to confirm these effects under real oral conditions.

## Data Availability

The data generated and analyzed during the current study are available from the corresponding author on reasonable request.
